# Screening for data clustering in multicenter studies: the residual intraclass correlation

**DOI:** 10.1186/1471-2288-13-128

**Published:** 2013-10-23

**Authors:** Laure Wynants, Dirk Timmerman, Tom Bourne, Sabine Van Huffel, Ben Van Calster

**Affiliations:** 1 KU Leuven Department of Electrical Engineering-ESAT, STADIUS Center for Dynamical Systems, Signal Processing and Data Analytics, Leuven, Belgium; 2 KU Leuven iMinds Future Health Department, Leuven, Belgium; 3 KU Leuven Department of Development and Regeneration, Leuven, Belgium; 4 Department of Obstetrics and Gynaecology, University Hospitals Leuven, Leuven, Belgium; 5 Early pregnancy and Gynaecological Ultrasound unit, Queen Charlottes and Chelsea Hospital, Du Cane Road, London, UK; 6 Institute of Reproductive and Developmental Biology, Imperial College, London, UK

## Abstract

**Background:**

In multicenter studies, center-specific variations in measurements may arise for various reasons, such as low interrater reliability, differences in equipment, deviations from the protocol, sociocultural characteristics, and differences in patient populations due to e.g. local referral patterns. The aim of this research is to derive measures for the degree of clustering. We present a method to detect heavily clustered variables and to identify physicians with outlying measurements.

**Methods:**

We use regression models with fixed effects to account for patient case-mix and a random cluster intercept to study clustering by physicians. We propose to use the residual intraclass correlation (RICC), the proportion of residual variance that is situated at the cluster level, to detect variables that are influenced by clustering. An RICC of 0 indicates that the variance in the measurements is not due to variation between clusters. We further suggest, where appropriate, to evaluate RICC in combination with R^2^, the proportion of variance that is explained by the fixed effects. Variables with a high R^2^ may have benefits that outweigh the disadvantages of clustering in terms of statistical analysis. We apply the proposed methods to a dataset collected for the development of models for ovarian tumor diagnosis. We study the variability in 18 tumor characteristics collected through ultrasound examination, 4 patient characteristics, and the serum marker CA-125 measured by 40 physicians on 2407 patients.

**Results:**

The RICC showed large variation between variables: from 2.2% for age to 25.1% for the amount of fluid in the pouch of Douglas. Seven variables had an RICC above 15%, indicating that a considerable part of the variance is due to systematic differences at the physician level, rather than random differences at the patient level. Accounting for differences in ultrasound machine quality reduced the RICC for a number of blood flow measurements.

**Conclusions:**

We recommend that the degree of data clustering is addressed during the monitoring and analysis of multicenter studies. The RICC is a useful tool that expresses the degree of clustering as a percentage. Specific applications are data quality monitoring and variable screening prior to the development of a prediction model.

## Background

In clinical research, multicenter consortia are rapidly gaining popularity. Recruiting patients from a broad range of settings yields a representative sample, and simultaneously patient recruitment times can be reduced [1,2]. This is especially appealing when studying rare diseases. A complication that arises from multicenter data collection is data clustering: two patients from the same center have more similarities than two patients from different centers. Most of the common statistical analysis techniques assume independent data. This assumption is violated in clustered data. As a consequence, studies may be underpowered. Estimates of standard errors can be incorrect, and hence the type-I error rate is too. Confidence intervals will often be too narrow. Furthermore, regression estimates may be biased due to confounding of covariates with center, or diluted due to lack of agreement in measurements by different physicians. If clustering is not accounted for during analysis, research conclusions can be misleading [3].

We believe that in many studies it is useful to investigate clustering in detail in order to detect problems or understand the data structure. Important causes of clustering are non-random differences in the measurements taken by different physicians recruiting in the contributing centers. Intraclass correlations (ICC) have traditionally been used to study interrater reliability. The ICC is commonly defined as the correlation between two quantitative measurements made by different judges or raters for the same measurement object [4,5]. However, in the context of multicenter studies, it has some drawbacks. First, it requires at least two raters measuring variables for the same patient. Since most multicenter studies are not designed for studying interrater reliability, this would require additional efforts when designing the study and collecting the data. It is often impractical and cost-intensive to have physicians from different centers examining the same patients. Second, interrater reliability studies do not uncover all relevant forms of data clustering in multicenter studies. Besides subjectivity of measurements, there are various other reasons for non-random differences across physicians. Differences in equipment or equipment settings may give rise to clustering, as well as deviations from the study protocol. Regional differences across centers may cause data clustering via sociocultural characteristics of patients or procedures. It is for example well documented that the perception and expression of pain is related to ethnicity [6]. Non-random differences in measurements across physicians may also occur because of differences in their patient populations due to e.g. local referral patterns.

An alternative formulation of the ICC does not require measurements by multiple physicians for the same patient. Instead, it requires the assessment of multiple patients per physician, which is more in line with typical data collection in multicenter studies. In this set-up, the ICC is defined as the correlation between any two measurements made by the same physician, or equivalently the amount of variance in the measurements that is located at the level of the physician. As a consequence, it is able to detect various forms of data clustering. Hence, it can be used for data quality control, as recently demonstrated by Guthrie et al. [7], or as a screening step before selecting variables to develop a prediction model. A drawback of this formulation of the ICC is that it does not acknowledge that physicians may systematically record lower or higher measurements because of different patient populations.

In this work we present mixed effects models to study clustering while simultaneously taking case-mix differences across physicians into account. We use the residual intraclass correlation (RICC), an extension of the ICC, to quantify the degree of clustering. We also relate the RICC to the proportion of explained variance by partitioning the total variance into error variance at the patient and physician level, and explained variance. We demonstrate these concepts on a multicenter dataset collected for the development and validation of a prediction model for preoperative ovarian and tubal tumor diagnosis.

## Methods

### A mixed effects model

Suppose a variable *Z*_*ij*_ was measured in *N* patients by *J* physicians (clusters), *i* = 1,…, *N* and j = 1,…, *J*. *Z*_*ij*_ could be, for example, tumor size or a score to quantify blood flow. To account for data clustering, we consider a mixed effects model of *Z*_*ij*_ by incorporating a random intercept *a*_*j*_. This recognizes that the average measured value may systematically vary from physician to physician:

(1) $$\begin{array}{l} Z_{\mathrm{ij}}=\alpha +a_{j}+e_{\mathrm{ij}}\\ a_{j}\sim N\left(0,\tau_{\mathrm{intercept}}^{2}\right)\\ e_{\mathrm{ij}}\sim N\left(0,\sigma_{\mathrm{error}}^{2}\right). \end{array}$$

The random intercept at the physician level and the random error term at the patient level are assumed to have a normal distribution with mean zero and variance $\tau_{\mathrm{intercept}}^{2}=\mathrm{var}\left(a_{j}\right)$ and $\sigma_{\mathrm{error}}^{2}$, respectively. When *Z*_*ij*_ is a nominal or ordinal variable, model (1) can easily be reformulated as a generalized linear mixed model. Throughout this research, we use the logit link function in logistic regression models for dichotomous variables and proportional odds models for ordinal variables. Consequently, the error terms have a logistic distribution with σerror2=π23≈3.29[8].

### The intraclass correlation coefficient

The crude amount of clustering in *Z*_*ij*_ can be expressed as the proportion of variance at the cluster level. The total variance in *Z*_*ij*_ can be split up into the variance at the patient level ($\sigma_{\mathrm{error}}^{2}$) and variance at the physician level ($\tau_{\mathrm{intercept}}^{2}$). The intraclass correlation (ICC) is defined as

(2) $$\mathrm{ICC}=\frac{\tau_{\mathrm{intercept}}^{2}}{\tau_{\mathrm{intercept}}^{2}+\sigma_{\mathrm{error}}^{2}}.$$

The patient populations (case-mix) often vary between clusters. In this case a more refined metric is required, as described below.

### Patient case-mix

Patient case-mix can account for a substantial part of the between-physician variance. For example, in a diagnostic accuracy study the prevalence of the disease under study may differ across physicians. In that case, the true disease status of patient *i* seen by physician *j* can be included as an explanatory variable in the model of *Z*_*ij*_ (1):

(3) $$\begin{array}{l} Z_{\mathrm{ij}}=\alpha +a_{j}+\beta \times \mathrm{diseas}e_{\mathrm{ij}}+e_{\mathrm{ij}}\\ a_{j}\sim N\left(0,\tau_{\mathrm{intercept}}^{2}\right)\\ e_{\mathrm{ij}}\sim N\left(0,\sigma_{\mathrm{error}}^{2}\right). \end{array}$$

The true disease status will usually be represented by a dummy variable indicating presence or absence of the disease, although an extension to multiple dummies to describe the true disease status in more than two categories is straightforward. In what follows, we use disease as the case-mix factor, although in other applications different variables may be more relevant.

Additional explanatory patient-level factors can be added to the model if necessary. Patient-level variables that are related to *Z*_*ij*_ and have unequal distributions across clusters potentially explain clustering, and are thus most relevant to include. Patient-level factors influencing *Z*_*ij*_ other than the factors included in the model for Z_*ij*_ are regarded as disturbances captured by the error term.

### The residual intraclass correlation coefficient

The total variance of *Z*_*ij*_ can now be split up into a part that is accounted for by the explanatory variables ($\sigma_{\mathrm{LP}}^{2}$), an unexplained part at the patient level ($\sigma_{\mathrm{error}}^{2}$) and an unexplained part at the physician level ($\tau_{\mathrm{intercept}}^{2}$). $\sigma_{\mathrm{LP}}^{2}$ is the variance of the linear predictor, excluding the random intercepts. In model (3) this is var(*β* × *disease*_*ij*_). R^2^ is the proportion of variance in *Z*_*ij*_ accounted for by the explanatory variables:

(4) $$R^{2}=\frac{\sigma_{\mathrm{LP}}^{2}}{\sigma_{\mathrm{LP}}^{2}+\tau_{\mathrm{intercept}}^{2}+\sigma_{\mathrm{error}}^{2}}.$$

A higher R^2^ indicates that the variable is less influenced by disturbances at the physician and patient level. The proportion of total variance at the physician level is labelled as the 'variance partitioning coefficient’ (VPC):

(5) $$\mathrm{VPC}=\frac{\tau_{\mathrm{intercept}}^{2}}{\sigma_{\mathrm{LP}}^{2}+\tau_{\mathrm{intercept}}^{2}+\sigma_{\mathrm{error}}^{2}}.$$

Theoretically VPC can take values between 0 and 1, but 1 will only be reached if there is no residual variance at the patient level ($\sigma_{\mathrm{error}}^{2}=0$), and the explanatory variables have the same value for all patients or all have regression coefficients of zero ($\sigma_{\mathrm{LP}}^{2}=0$). The sum of the R^2^, the VPC and the proportion unexplained variance at the patient level equals one. Since its magnitude depends on $\sigma_{\mathrm{LP}}^{2}$, the VPC is difficult to interpret as a measure of the degree of clustering.

The residual intraclass correlation (RICC) is the residual correlation in the measurements between any two patients seen by the same physician, after the effects of the explanatory variables have been taken into account [8]. Equivalently, it is the proportion of the residual variance situated at the physician level:

(6) $$\mathrm{RICC}=\frac{\tau_{\mathrm{intercept}}^{2}}{\tau_{\mathrm{intercept}}^{2}+\sigma_{\mathrm{error}}^{2}}.$$

Note that $\sigma_{\mathrm{LP}}^{2}$ is not part of the denominator. The RICC reaches a maximum value of 1 when all of the variance in *Z*_*ij*_ that is not explained by the explanatory variables, is situated at the physician level. If the residual variance is only situated at the level of the patient, the RICC reaches a minimum value of 0, and there are no between-physician differences. This makes the RICC a pure measure of clustering and an easy to interpret screening tool. However, it may be useful in certain applications to include R^2^ in the evaluation of the RICC and in the subsequent decision to investigate the cause of clustering or to exclude variables from further analysis. Variables with a high proportion of explained variance (R^2^) may have benefits that outweigh the disadvantages of clustering.

### Elaborating the mixed effects model

An often overlooked assumption underlying a mixed model is that random terms should be uncorrelated with explanatory variables [3,8]. It is unlikely that this assumption always holds in multicenter studies. For example, when we are interested in clustering of patients with a history of cancer within physicians, the variable 'history of cancer’ is the dependent variable (*Z*_*ij*_) in model (3) above. Physicians specialized in cancer treatment may regularly encounter patients with a history of cancer, a risk factor for tumor malignancy when the patient presents with a new mass. Therefore, they will have high random intercepts (*a*_*j*_) compared to other physicians. At the same time these physicians may also encounter malignant tumors more often than other physicians. Complex referral patterns exist in clinical practice, which may cause patients with suspected recurrent cancers and other highly suspicious masses to be referred to specialized physicians. Hence, there is a correlation between the random intercept and the tumor type. If we include tumor type to account for differences in patient case-mix (*disease*_*ij*_), the regression coefficient for this variable (*β*) is a joint estimate reflecting not only the association of cancer history with tumor type, but also the association of the prevalence of patients with a cancer history with the prevalence of malignant tumors at the cluster level [8]. These between- and within physician associations cannot be separated, which is problematic since they can differ in strength and even have opposite signs.

The solution is to add physician-level information on their patients’ true disease status to the model, in order to obtain an unbiased estimate of the within-physician effect of tumor type [8]. When the disease status is a dichotomous variable with categories indicating presence or absence of the disease, this is simply the prevalence of the disease for physician *j*. When disease status has *k* categories, *k*-1 variables need to be constructed, each indicating the prevalence of one of the categories for physician *j*. When disease status is a continuous variable, the physician-specific average should be included. Model (3) can be extended as follows:

(7) $$\begin{array}{l} Z_{\mathrm{ij}}=\alpha +a_{j}+\beta_{1}\times \mathrm{disease}\_\mathrm{prevalenc}e_{j}+\beta_{2}\times \mathrm{diseas}e_{\mathrm{ij}}+e_{\mathrm{ij}}\\ a_{j}\sim N\left(0,\tau_{\mathrm{intercept}}^{2}\right)\\ e_{\mathrm{ij}}\sim N\left(0,\sigma_{\mathrm{error}}^{2}\right). \end{array}$$

The constant term for physician *j* now equals $\alpha_{j}^{*}=\alpha +a_{j}+\beta_{1}\times \mathrm{disease}\_\mathrm{prevalenc}e_{j}$ and model (7) can be rewritten as

(8) $$\begin{array}{l} Z_{\mathrm{ij}}=\alpha_{j}^{*}+\beta_{2}\times \mathrm{diseas}e_{\mathrm{ij}}+e_{\mathrm{ij}}.\\ \alpha_{j}^{*}\sim N\left(\bar{\alpha_{j}^{*}},\tau_{\mathrm{intercept}}^{2}\right)\\ e_{\mathrm{ij}}\sim N\left(0,\sigma_{\mathrm{error}}^{2}\right). \end{array}$$

$$\bar{\alpha_{j}^{*}}=\alpha +\beta_{1}\times \bar{\mathrm{disease}\_\mathrm{prevalenc}e_{j}}$$

is the overall intercept, around which the $\alpha_{j}^{*}$ vary with a variance $\tau_{\mathrm{intercept}}^{2}$, which now equals

$$\mathrm{var}\left(\alpha_{j}^{*}\right)=\mathrm{var}\left(a_{j}\right)+\beta_{1}^{2}\times \mathrm{var}\left(\mathrm{disease}\_\mathrm{prevalenc}e_{j}\right)+2\beta_{1}\times \mathrm{cov}\left(a_{j},\mathrm{disease}\_\mathrm{prevalenc}e_{j}\right).$$

Finally, analogous to the fact that patient information can explain a part of the between-physician variance (cf. $\mathrm{var}\left(\alpha_{j}^{*}\right)$), so can information at the physician level. Therefore it is possible to include physician-level explanatory variables, such as their workplace (e.g. regional hospital or tertiary center):

(9) $$\begin{array}{l} Z_{\mathrm{ij}}=\alpha_{j}^{*}+\beta_{2}\times \mathrm{diseas}e_{\mathrm{ij}}+\gamma \times \mathrm{physician}\_\mathrm{characteristi}c_{j}+e_{\mathrm{ij}}.\\ \alpha_{j}^{*}\sim N\left(\bar{\alpha_{j}^{*}},\tau_{\mathrm{intercept}}^{2}\right)\\ e_{\mathrm{ij}}\sim N\left(0,\sigma_{\mathrm{error}}^{2}\right). \end{array}$$

Note that the variance of the linear predictor, $\sigma_{\mathrm{LP}}^{2}$, now equals var(*β*_2_ × *disease*_*ij*_ + *γ* × *physician* _ *characteristic*_*j*_).

### Estimation

In practice, the variance terms $\tau_{\mathrm{intercept}}^{2}$, $\sigma_{\mathrm{error}}^{2}$ and $\sigma_{\mathrm{LP}}^{2}$ can be estimated using any standard statistical package that allows for the fitting of mixed effect models. We have used SAS software (version 9.3, SAS Institute, Cary, NC, USA) for all computations. A SAS macro for the computation of the RICC, based on the mixed and glimmix procedures, has been included in (Additional file 1).

$$\tau_{\mathrm{intercept}}^{2}$$

and $\sigma_{\mathrm{error}}^{2}$ can be estimated by first estimating full model (7) to obtain ${\hat{\beta }}_{2}$, and subsequently fitting a random intercept model for *Z*_*ij*_ without explanatory variables but with ${\hat{\beta }}_{2}\times \mathrm{diseas}e_{\mathrm{ij}}$ as an offset variable. If physician-level explanatory variables are included, full model (9) including the physician-level fixed effect is fitted. Subsequently a random intercept model for *Z*_*ij*_ is fitted with ${\hat{\beta }}_{2}\times \mathrm{diseas}e_{\mathrm{ij}}$ as an offset variable and the physician-level effect as explanatory variable to re-estimate *γ*. The resulting variance of the random intercept is the estimated variance of the random intercepts $\alpha_{j}^{*}$, $\tau_{\mathrm{intercept}}^{2}$, while the residual variance is the estimated variance of the error term, $\sigma_{\mathrm{error}}^{2}$. If *Z*_*ij*_ is a dichotomous or ordinal variable and generalized linear models are fitted using the logit transformation as a link function, the error terms have a logistic distribution with σ2=π23≈3.29[8].

The estimation of $\sigma_{\mathrm{LP}}^{2}$ was chosen to correspond to standard ways of computing R^2^ in linear and generalized linear mixed models [8]. To obtain the explained variance for a continuous *Z*_*ij*_, an empty random intercept model, i.e. without fixed patient or physician level explanatory variables, was fitted. The resulting estimated variance at physician and patient level was added together to obtain an estimate of the total variance. The explained variance ($\sigma_{\mathrm{LP}}^{2}$) can subsequently be estimated by subtracting ${\hat{\tau }}_{\mathrm{intercept}}^{2}$ and ${\hat{\sigma }}_{\mathrm{error}}^{2}$ from the estimated total variance. In the case of an ordinal or dichotomous predictor, $\sigma_{\mathrm{LP}}^{2}$ was computed directly from the data, obtaining the linear predictor by multiplying the explanatory variables with the regression coefficient estimates from full model (7) or (9), as appropriate.

### Confidence statements

90% bootstrap confidence intervals were computed using the percentile method. Since data are clustered, bootstrap resampling was performed at the physician level, including all patients for each sampled physician [9].

### Random intercept plots to identify outlying physicians

The random intercepts obtained when fitting model (8) or (9) can be plotted to identify physicians with measurements for *Z*_*ij*_ that are unusually large or small, given the explanatory variables and compared to measurements by the average physician. Note that estimated random intercepts are shrunken towards zero, and that shrinkage increases as the number of patients seen by physician *j* decreases. Comparative standard errors are used to test whether the physician-specific random intercept is significantly different from zero [10]. We used the false discovery rate method to account for multiple testing [11].

### Data

We illustrate the described techniques on data from the International Ovarian Tumor Analysis (IOTA) group containing clinical and ultrasound information on 2407 patients with ovarian or tubal tumors, prospectively collected between 2002 and 2007 by 40 physicians from 19 hospitals in 8 countries. The data was collected to develop and validate clinical prediction models for the diagnosis of ovarian and tubal tumors [12-16]. University Hospitals Leuven is the coordinating center of the IOTA studies. The study protocols for the collection of the data were approved by the Ethics Committee of the University Hospitals Leuven ('Commissie Medische Ethiek’) and by the local Ethics Committee at each recruitment center.

We focused on clustering at the physician level. Additional clustering of physicians in hospitals was not taken into account during analysis because in 10 of the hospitals data was collected by only 1 physician, whereas for the other 9 hospitals there was 1 principal investigator collecting the vast majority of the data. The RICC was used to study between-physician variance for 18 variables collected through an ultrasound examination, 4 patient characteristics and the level of serum marker CA125. To account for differences in case-mix, mixed models were developed, with tumor histology (benign, borderline, primary invasive or metastatic invasive) as the explanatory variable. After patients obtained surgery, all excised tissues were sampled for histological examination at the local center. Tumor histology was then determined according to the World Health Organization classification [17]. Ultrasound machine quality (high end, medium end and low end machines) was included in a second step as a physician-level characteristic to further explain differences between physicians in ultrasound measurements. Discussions with clinical experts were held to reveal to reveal the likely causes of the high interphysician variability of certain measurements. Additionally, a limited survey was conducted among physicians with unusually high or low random intercepts for highly clustered variables. The survey included questions on the measurement or registration for each of these variables.

## Results

The median number of patients per physician is 9 (mean 60, IQR 2 to 82.5, range 1 to 509). 18 of 40 physicians have seen less than 5 patients, while 16 have seen more than 30. In total 72.1% of patients had a benign tumor, while in 5.4% of patients the tumor was borderline, in 19.4% it was primary invasive and in 3.2% it was a metastatic tumor in the ovary. However, among physicians with more than 5 patients, the prevalence of benign, borderline, invasive and metastatic tumors per physician varied from 28.6 to 91.9%, 0 to 20.0%, 0 to 71.4% and 0 to 7.5%, respectively, indicating considerable case-mix differences.

**Figure 1:**
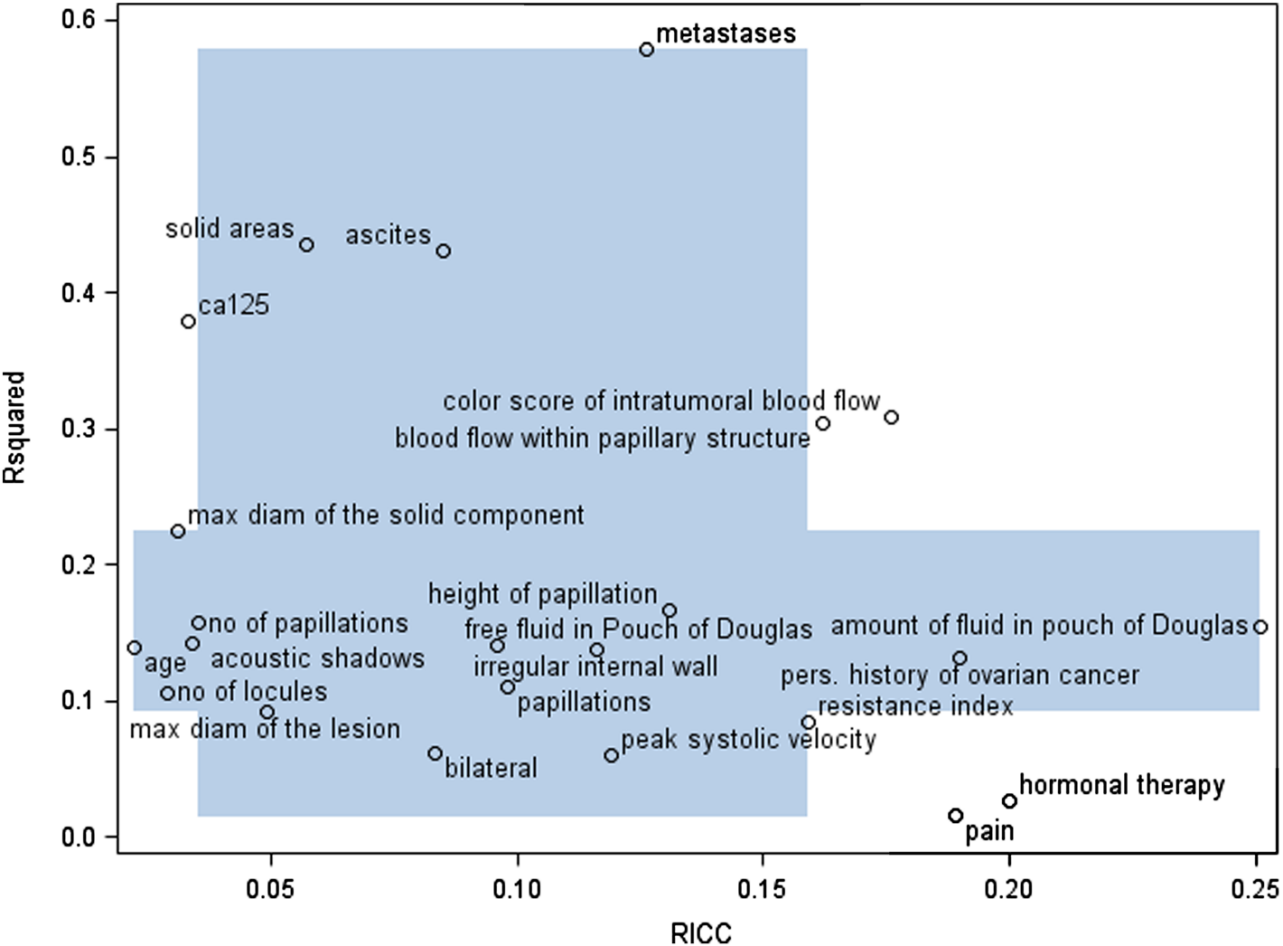
**Plot of RICC versus R**^**2**^**.** Dark bands indicate the interquartile range for RICC and R^2^.

The RICC showed considerable variation between variables (Table 1, Figure 1). Patient age had the lowest level of clustering (RICC 2.2%, 90% CI 0.9-3.7%). Six other variables had an RICC below 5% (number of locules, maximum diameter of the solid component, serum CA-125, acoustic shadows, number of papillations, and maximum lesion diameter). On the other hand, for the amount of fluid in the pouch of Douglas, 25.1% (90% CI 6.7-32.1%) of the residual variance was due to between-physician differences. For current use of hormonal therapy this was 20.0% (90% CI 10.6-31.2%). Five other variables had an RICC above 15% (personal history of ovarian cancer, pelvic pain during examination, color score of intratumoral blood flow, presence of papillations with detectable flow, and the resistance index). Of these seven variables with an RICC above 15%, the amount of variability accounted for by tumor type varies from 1.5% (90% CI 0.9-9.4%) for pelvic pain and 2.6% (90% CI 1.3-9.0%) for current use of hormonal therapy to 30.4% (90% CI 25.6-41.2%) for presence of papillations with detectable flow and 30.9% (90% CI 24.9-36.3%) for color score of intratumoral flow.

**Table 1:** Variance partitioning of ultrasound measurements and patient characteristics

**Variable**	**n**	**Coefficient of variation/Prevalence**	**VPC**	**R**^**2**^	**RICC**
Patient age (years)	2407	0.4	1.9%	13.9%	2.2%
			[0.8% to 3.3%]	[10.6% to 17.4%]	[0.9% to 3.7%]
Number of locules (ordinal)	1997^c^	0.8	2.6%	10.6%	2.9%
			[0.5% to 4.4%]	[6.7% to 14.2%]	[0.6% to 4.9%]
Maximum diameter of the solid component (mm, log transformed)	1160^b^	0.2	2.4%	22.4%	3.1%
			[0.0% to 5.7%]	[17.4% to 28.9%]	[0.0% to 7.3%]
Serum CA125 (IU/L, log transformed)	1827^a^	0.4	2.0%	37.8%	3.3%
			[0.8% to 4.3%]	[32.8% to 41.7%]	[1.3% to 6.8%]
Acoustic shadows (yes/no)	2407	13.7%	3.0%	14.2%	3.4%
			[0.0% to 5.0%]	[8.0% to 72.1%]	[0.0% to 5.9%]
Number of papillations (ordinal)	468^d^	0.6	2.9%	15.7%	3.5%
			[0.0% to 5.5%]	[13.6% to 23.8%]	[0.0% to 6.6%]
Maximum lesion diameter (mm, log transformed)	2406^e^	0.1	4.5%	9.2%	4.9%
			[1.0% to 10.1%]	[5.9% to 11.4%]	[1.1% to 11.0%]
Presence of solid components (yes/no)	2407	48.2%	3.2%	43.5%	5.7%
			[1.0% to 4.4%]	[39.8% to 73.5%]	[2.3% to 8.4%]
Bilateral (yes/no)	2407	16.6%	7.8%	6.1%	8.3%
			[0.0% to 30.0%]	[3.6% to 8.2%]	[0.0% to 31.6%]
Ascites (yes/no)	2407	10.0%	4.9%	43.0%	8.5%
			[0.0% to 9.5%]	[34.5% to 49.3%]	[0.0% to 15.8%]
Free fluid in pouch of Douglas (yes/no)	2407	25.4%.	8.3%	14.0%	9.6%
			[2.2% to 13.3%]	[11.1% to 17.5%]	[2.6% to 15.2%]
Presence of papillations (yes/no)	2407	19.6%	8.7%	11%	9.8%
			[3.7% to 12.9%]	[7.4% to 14.9%]	[4.1% to 14.8%]
Irregular internal wall (yes/no)	2407	38.5%	10.0%	13.8%	11.6%
			[2.7% to 16.7%]	[11.6% to 16.7%]	[3.1% to 19.5%]
Peak systolic velocity (cm/s, log transformed)	1432^g^	0.3	11.2%	5.9%	11.9%
			[5.3% to 16.3%]	[2.4% to 10.8%]	[6.0% to 16.9%]
Metastases (yes/no)	1457^f^	10.7%	5.3%	57.9%	12.6%
			[0.2% to 8.4%]	[52.9% to 95.3%]	[1.3% to 21.1%]
Height of papillation (mm, log transformed)	468^d^	0.3	10.9%	16.7%	13.1%
			[0.0% to 28.2%]	[6.3% to 25.5%]	[0.0% to 31.7%]
Resistance index	1432^g^	0.3	14.5%	8.4%	15.9%
			[1.7% to 23.6%]	[4.6% to 13.5%]	[1.9% to 26.5%]
Papillations with detectable blood flow (yes/no)	468^d^	47.4%	11.3%	30.4%	16.2%
			[2.8% to 17.1%]	[25.6% to 41.2%]	[4.4% to 23.8%]
Color score of intratumoral blood flow (ordinal)	2407	0.5	12.2%	30.9%	17.6%
			[3.6% to 22.6%]	[24.9% to 36.3%]	[5.6% to 30.1%]
Pelvic pain during examination (yes/no)	2407	19.1%	18.6%	1.5%	18.9%
			[6.6% to 26.5%]	[0.9% to 9.4%]	[9.8% to 27.1%]
Personal history of ovarian cancer (yes/no)	2407	1.6%	16.5%	13.1%	19.0%
			[0.0% to 25.3%]	[10.3% to 72.8%]	[0.0% to 30.3%]
Current use of hormonal therapy (yes/no)	2407	12.7%	19.5%	2.6%	20.0%
			[10.0% to 30.4%]	[1.3% to 9.0%]	[10.6% to 31.2%]
Amount of free fluid in pouch of Douglas (mm, log transformed)	616^h^	0.3	21.2%	15.5%	25.1%
			[5.1% to 28.4%]	[9.8% to 21.7%]	[6.7% to 32.1%]

Percentage of the total variance at the sonographer level (VPC) (% [90 CI]), percentage of the total variance explained by tumour type (R^2^) (% [90 CI]) and the residual intraclass correlation (RICC) (% [90 CI]). ^a^Measurement of CA125 was not obligatory; ^b^If at least one solid component is present; ^c^If there is at least one locul; ^d^In case of the presence of a papillary structure; ^e^One influential outlier removed (measurement error); ^f^Assessment of presence of metastases was not obligatory; ^g^If there is intratumoural blood flow; ^h^If there is fluid in the pouch of Douglas.

The VPC varied from 1.9% (90% CI 0.8% to 3.3%) for patient age to 21.2% (90% CI 5.1% to 28.4%) for the amount of fluid in the pouch of Douglas. VPC and RICC were similar for most variables. However, when the tumor type accounts for a large proportion of the total variance (R^2^ is high), the VPC was by definition considerably smaller than the RICC. For example, 12.6% of the residual variance of the presence of metastases was due to between-physician differences, but the VPC was only 5.3%: of the total variance in metastases 57.9% was accounted for by tumor type.

Taking the ultrasound machine quality into account yielded a considerable reduction in the RICC of Doppler blood flow indices such as peak systolic velocity (reduction in RICC 4.0%, 90% CI -0.5% to 9.4%) and resistance index (reduction in RICC 5.6%, 90% CI -1.3% to 19.2%) (Table 2). The measurements of blood flow depend on the quality of the ultrasound machine, with higher quality machines giving more sensitive measurements. The explanatory power of ultrasound machine quality was moderate at best for the other indicators.

**Table 2:** Effect of ultrasound machine quality on differences between physicians

**Variable**	**n**	**Reduction in RICC**
Number of locules (ordinal)	1997^c^	0.3%
		[-0.1% to 1.2%]
Maximum diameter of the solid component (mm, log transformed)	1160^b^	-0.5%
		[-1.1% to 1.1%]
Acoustic shadows (yes/no)	2407	-0.4%
		[-3.6% to 0.9%]
Number of papillations (ordinal)	468^d^	3.1%
		[-0.0% to 6.2%]
Maximum lesion diameter (mm, log transformed)	2406^e^	0.2%
		[-0.6% to 2.8%]
Presence of solid components (yes/no)	2407	0.7%
		[-0.3% to 2.6%]
Bilateral (yes/no)	2407	-1.2%
		[-3.7% to 2.1%]
Ascites (yes/no)	2407	3.6%
		[-0.8% to 10.8%]
Free fluid in pouch of Douglas (yes/no)	2407	0.6%
		[-0.5% to 3.1%]
Presence of papillations (yes/no)	2407	2.5%
		[0.2% to 6.3%]
Irregular internal wall (yes/no)	2407	1.0%
		[-0.5% to 4.1%]
Peak systolic velocity (cm/s, log transformed)	1432^g^	4.0%
		[-0.5% to 9.4%]
Metastases (yes/no)	1457^f^	0.9%
		[-1.3% to 6.5%]
Height of papillation (mm, log transformed)	468^d^	0.1%
		[-2.9% to 8.8%]
Resistance index	1432^g^	5.6%
		[-1.3% to 19.2%]
Papillations with detectable blood flow (yes/no)	468^d^	2.7%
		[-1.5% to 12.8%]
Color score of intratumoral blood flow (ordinal)	2407	-0.4%
		[-2.2% to 1.5%]
Amount of free fluid in pouch of Douglas (mm, log transformed)	616^h^	0.8%
		[-0.9% to 3.9%]

Difference in RICC [90% CI]. ^b^If at least one solid component is present; ^c^If there is at least one locul; ^d^In case of the presence of a papillary structure; ^e^One influential outlier removed (measurement error); ^f^Assessment of presence of metastases was not obligatory; ^g^If there is intratumoural blood flow; ^h^If there is fluid in the pouch of Douglas.

**Figure 2:**
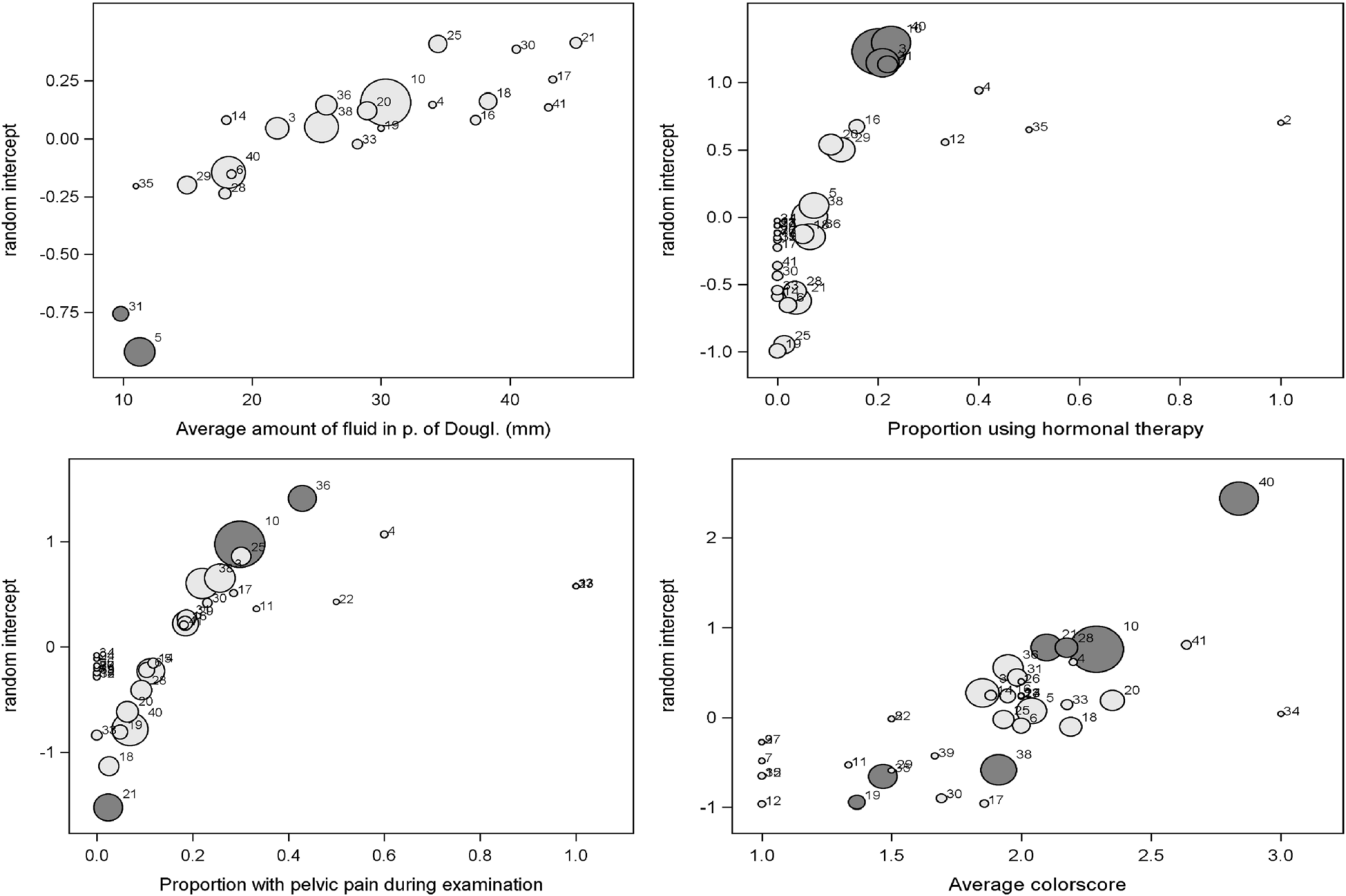
**Random physician intercept plots.** From left to right and from top to bottom: amount of free fluid in the pouch of Douglas (mm), current use of hormonal therapy (yes/no), pelvic pain during examination (yes/no), color score of intratumoral blood flow (ordinal, 1 no blood flow, 2 minimal blood flow, 3 moderate blood flow, 4 very strong blood flow). X-axis: average measure or prevalence per physician. Bubble size represents the number of patients per physician. Dark bubbles: physicians with a random intercept different from zero (adjusted p-value < 0.05 after correction for multiple testing using the false discovery rate method).

To detect which physicians have systematically higher or lower measurements than the average physician, random intercept plots were constructed showing the physician’s central tendency (mean or proportion) on the x-axis and the random intercept on the y-axis (Figure 2). There was considerable interphysician variability in the measurements of the amount of fluid in the pouch of Douglas, with average measurements per physician ranging from 10 mm to 45 mm. Taking patient case-mix into account, two physicians were identified as outlying with lower measurements than the average physician. Physician 5 positioned his patients horizontally on a flat bench while measuring the fluid, which could explain the low measurements. Physician 31 used an examination table with stirrups, on which the patient was placed in the supine position. However, the gradient of the patient’s trunk, which is most likely to influence the measured amount of fluid in the pelvis, is unknown. Hence, we could not determine why this physician obtains remarkably low measurements.

Use of hormonal therapy at the time of the ultrasound examination was between zero and ten percent for most physicians (Figure 2). Given patient case-mix, four physicians were identified as outlying with higher rates of hormonal therapy use than the average physician, as indicated by the physicians’ positive random intercepts. Three of them were from centers in Belgium and Sweden, both countries in which hormonal therapy is more frequently prescribed than in other countries, such as Italy. The survey among physicians revealed that the outliers had given their patients various examples when asking them about hormonal therapy use, which could have contributed to the large numbers of patients with hormonal therapy use that they registered. The survey further revealed that physicians do not fully agree on what they consider to be hormonal therapy.

The interphysician variability in patients’ experience of pelvic pain during examination could not be explained by the physician’s examination style, the type of probe that was used, or the prevalence of endometriomas or abscesses, which are known to be more painful than other tumors, especially if pressure is applied on these masses. It is likely that the registration of pain was subject to the empathy of the physician on the one hand, and the pain threshold and inclination of the patient to express pain on the other. The latter may vary from person to person and additionally may also be country-dependent [6].

Eight physicians were detected as outliers for the color score of intratumoral blood flow, five with high and three with low values, which may partly be explained by the use of color or power Doppler ultrasonography by different examiners. The survey among outlying physicians included five images of ovarian masses, which had to be rated. People with a tendency to give high scores to the survey images also tended to have higher random intercepts, and vice versa, indicating the subjectivity of color scores.

## Discussion

### Overview

In this paper we describe a novel use of the RICC as a useful tool to screen variables for clustering in multicenter studies. It expresses the degree of clustering as a percentage of the residual variance. Mixed models, allowing control for patient- and cluster-level fixed effects, are used to estimate the three parts of the total variance: variance explained by the fixed effects (R^2^), residual variance at the patient level and residual variance at the cluster level.

Depending on the context, it can be useful to take R^2^ into account when evaluating the degree of clustering, comparable to a cost-benefit analysis (Figure 1). The 'cost’ (RICC) is the degree of clustering which complicates statistical analysis and may necessitate efforts to detect and remove causes of clustering. The 'benefit’ is the extent to which a variable is related to the fixed effects, for example the disease of interest. A higher R^2^ indicates that the variable is less influenced by disturbances at the physician and patient level. For a variable with a high RICC but low R^2^, it may not be worth the effort to alleviate the amount of clustering and the variable may be excluded from further data collection or analysis. For variables with a high RICC and R^2^, e.g. color score of intratumoral blood flow in our case study, it can be worthwhile to investigate the cause of the clustering. Careful consideration should be given to whether the covariance with the fixed effects outweighs the disadvantages of data clustering.

### Applications

The proposed methods can be used for quality control when data collection in a large multicenter study is ongoing. In this way, problems with data clustering can be identified and remedied in a timely fashion. Another application is in model development, for example, of clinical prediction models [18]. Some researchers state that, before a variable is considered for inclusion in a prediction model, its interrater reliability should be assessed [19]. Others argue that this is superfluous because the effect of unreliable measurements in multivariable models will be diluted, i.e. self-penalization of unreliable predictors. In our opinion it is preferable to screen for various other forms of data clustering as part of the data analysis in a multicenter study as well. This is discussed in more detail in the next section.

Note that, when disease status is included as a fixed effect, simultaneously inspecting R^2^ while screening for clustering can provoke univariate variable selection. This is generally not recommended, especially when the dataset available for model building is limited in size [20]. In our case study, there were 672 malignant tumors for 23 potential predictors, resulting in nearly 30 events per potential predictor, while guidelines propose 10 to 50 events per variable depending on the situation [18]. In case variable selection is required, sample size is an important determinant of appropriate selection procedures, and we advise to rely mainly on prior expert knowledge and multivariate selection procedures. Nonetheless, R^2^ can play a role when sample size permits and when it is part of a carefully designed selection procedure. When data-driven variable selection is not an issue, the use of R^2^ is not problematic.

In randomized trials, a fixed effect that should be taken into account when studying clustering is the treatment arm, unless clustering of baseline measurements is investigated. Therefore, the proposed methodology can be applied during an interim analysis of the treatment effect. Recent studies have stressed the importance of acknowledging clustering in randomized controlled trials [21,22].

Regarding the final statistical analysis of the data, cluster-adjusted statistical techniques should be considered for multicenter data. Mixed effects models do not make the assumption that observations are independent and have the additional advantage of providing cluster-specific predictions [8,23].

### Strengths and weaknesses

A strength of the proposed approach is that it can be applied to any regular multicenter dataset, as it does not require measurements by multiple physicians for the same patient. In addition, various forms of data clustering are captured, including systematic interrater disagreement, differences in measurement equipment or settings, deviations from the measurement protocol, sociocultural characteristics, and differences in patient populations due to e.g. local referral patterns. The consequence is that the cause of the clustering is not immediately clear. In our survey, the problem was complicated further due to the amount of time that has passed between data collection and the survey on measurements (six years), potentially yielding recall bias. Nonetheless, in order to alleviate clustering it is imperative to investigate the causes of clustering. For example, when variables are subjective or the measurement protocol is unclear, providing training or protocol adjustment may help. It is also useful to detect clustering caused by differences in the populations seen by physicians (e.g. local referral patterns), since it is not possible to build a generalizable prediction model when such heterogeneity is too large.

Note that RICC only detects systematic differences in measurements between physicians. In the case of subjectivity of measurements, random intercepts will only indicate which physicians give consistently higher or lower scores than the average physician. Random (within-physician) variability will contribute to the residual variance at the patient level. To study non-systematic differences, an interrater reliability study is needed in which two or more physicians investigate the same patients in order to detect whether physicians would give the same scores to the same patients [4,5].

There are two methodological difficulties associated with our approach. First, a relatively large amount of data is needed to reliably compute the variance at the cluster level. This problem is most pronounced for categorical variables. The amount of variance at the cluster level will often be underestimated, a problem that increases as the number of clusters decreases [24-26]. This also explains the observed width of the bootstrap confidence intervals in the case study. Second, similar to provider profiling studies [18], it is not always straightforward how to take patient case-mix into account. In this study, tumor type was categorized into four groups (benign, borderline, invasive cancer, metastatic cancer), but a more general or more detailed categorization could have been chosen as well. This decision should involve experts’ opinions. Relevant categories should be taken into account, but the number of categories must be low enough to guarantee reliable estimates. For our case study, it is known that pelvic pain during examination might be worse for endometriomata and abscesses. Therefore, we could have used endometriomata and abscesses as a fifth category. Doing so marginally increased the RICC from 18.9% to 19.4%. This implies that caution must be taken when comparing the RICC across studies, as results may vary depending on variable definitions or the choice of fixed effect factors. For time-to-event outcomes, disease status may be accounted for by using the cumulative baseline hazard and an event indicator as fixed effects in the mixed model, analogous to a suggested approach for missing data imputation in the time-to-event setting [27].

## Conclusions

Although performing multicenter studies enhances generalizability of results, we recommend that the clustered nature of collected data is acknowledged and investigated. The RICC is a useful tool that expresses the degree of clustering as a percentage. An advantage of the RICC is that it does not require repeated measurements on the same patients by various physicians. The observed degree of clustering may be decreased by adjusting the measurement protocol and providing training to physicians.

## Competing interests

The authors declare that they have no competing interests.

## Authors’ contributions

LW initiated the design of the study and the development of the methodology, carried out statistical analyses and drafted the manuscript. DT made the data available and participated in the design of the study and the interpretation of the results. TB participated in interpreting the results. SVH participated in the design of the study. BVC participated in the design of the study, the development of the methodology and helped to draft the manuscript. All authors revised and approved the final manuscript.

## Pre-publication history

The pre-publication history for this paper can be accessed here:

http://www.biomedcentral.com/1471-2288/13/128/prepub

## Supplementary Material

Additional file 1 SAS macro to perform the RICC analyses. Click here for file
