# Supplementary material for: Screening for data clustering in multicenter studies: the residual intraclass correlation
Source: BMC Med Res Methodol. 2013 Oct 23;13:128. doi: 10.1186/1471-2288-13-128 (PMC3819732; doi:10.1186/1471-2288-13-128)
Supplement: Additional file 1 — SAS macro to perform the RICC analyses. [file 1471-2288-13-128-S1.doc]

/* SAS macro to perform the RICC analyses*/

*

Laure Wynants

18/08/2013

The RICC gives the proportion of variance of a variable due to differences between clusters, adjusted for several fixed effects that explain the differences between clusters.

It is a useful tool to screen for problematic variables in a multicentre study. It allows subjects to be nested in clusters and does not require that two or more physicians judge the same subject.

As such it can be used to detect problems with systematic differences in average measurements by phycisicans, due to e.g. cultural or regional differences, subjectivity of variables, miscabilbration of measurement tools, deviations from the measurement protocol or unequal distributions of variables accross clusters due to local referral patterns.

The outcome of this macro includes:

1) the residual intraclass correlation (RICC): gives the proportion of residual variance in the variable, after controlling for fixed effects. This will be 1 if within clusters, all subjects with a given level for the fixed predictors get the same score.

residual_variance_cluster / (residual_variance_cluster + residual_variance_subject)

2) the proportion of variance at the cluster level (VPC): Gives the proportion of the toal variance in the variable that is situated at the cluster level. This will be 1 only if within clusters, all subjects get the same score. residual_variance_cluster / (residual_variance_cluster + residual_variance_subject + variance_associated_with_fixed_predictor);

* to use this script, indicate a dataset 'data', a variable 'score' (the variable for which you want to study clustering),variables 'predictor' (fixed effects to account for clustering),and a cluster indicator 'cluster';

*note: REML is used.

*note: indicate whether the score variable's type is continous (cont), ordinal (ord) or dichotomous (dich);

*within-effects of the predictor need to be separated from between-effects. predictor is the list of (dummy) variables used to control for casemix (usually disease status). After 'between', indicate the mean/proportion of each of these variables per cluster;

* with macro_pred you can control for macrovariables, i.e. variables at the cluster level that can explain part of the residual ICC or VPC;

**%MACRO** variance_partition(data=, score=, cluster=, predictor=, between=, macropred=, type=);

options spool serror symbolgen;

%let predictor= %UPCASE(&predictor);

%let between= %UPCASE(&between);

%let macropred= %UPCASE(&macropred);

*1. CONTINUOUS VARIABLE;

%IF &type=cont %THEN %DO;

proc mixed data=&data;

class &cluster ;

model &score= &predictor &between &macropred/ s; /* we first include both between and within effetcs,so we can separate them to prevent confounding*/

random &cluster;

ods output solutionf=fixed;

run;

data fixed;

set fixed;

effect=upcase(effect);

run;

%let n=%sysfunc(countw(&predictor)); /*now we will isolate the within effect so we can continue to work with only the within effect*/

%do var=**1** %to &n;

%let withinvar= %qscan (&predictor, &var);

data _null_;

set fixed;

if effect="&withinvar" then call symput ('estimate', estimate);

run;

data &data;

set &data;

coeff=&estimate;

part_var_&var = &withinvar * coeff;

run;

%end;

data &data;

set &data;

within=sum(of part_var_1-part_var_&n);

score_within=&score-within;/*consider the within effect as an offset*/

drop coeff part_var_1-part_var_&n;

run;

proc mixed data=&data;/* run the model to determine the residual variance at cluster and subject level*/

class &cluster;

model score_within=&macropred; /*we have included the within as an offset to take only this into account, and thus prevent confounding of within and between cluster effects*/

random intercept /sub=&cluster solution; /* the random intercept is computed, this captures also the between-effect now*/

ods output CovParms=cluster_resid solutionr=RI; /* write variances to cluster_resid*/

run;

data cluster_resid;

set cluster_resid;

model='full'; /* indicate that this is the residual variance by recognizing the full model, including a fixed predictor, was fitted*/

run;

proc mixed data=&data; /* run an empty model to determine the total variance at cluster and subject level*/

class &cluster ;

model &score=;

random intercept /sub=&cluster;

ods output CovParms=total_cluster_resid; /* write variances to total_cluster_resid*/

run;

data total_cluster_resid;

set total_cluster_resid;

model='empty'; /* indicate that this is the total variance by recognizing the empty model was fitted*/

run;

/* now we write the variance at the cluster and subject to a macro variable. We do this for the empty model and for the full model accounting for the predictor*/

data _null_;

set total_cluster_resid;

if CovParm="Intercept" then call symput ("var_tot_cluster", Estimate);

if CovParm="Residual" then call symput ("var_tot_res", Estimate);

run;

data _null_;

set cluster_resid;

if CovParm="Intercept" then call symput ("var_res_cluster", Estimate);

if CovParm="Residual" then call symput ("var_res_res", Estimate);

run;

/* we compute the variance coefficient to quantify the total variability*/

proc means data=&data;

var &score;

output out=VC mean=mean std=std;

run;

data vc;

set vc;

Vc=mean/std;

if _type_=**0** then call symput ("coeff_var", vc);

run;

/* we compute the variance partitioning measures of interest*/

data outcome;

variation_coefficient=&coeff_var;

Residual_variance_cluster=&var_res_cluster;

Residual_variance_subject=&var_res_res;

Total_variance_cluster=&var_tot_cluster;

Total_variance_subject=&var_tot_res;

Total_variance=Total_variance_subject+Total_variance_cluster;

Variance_explained=Total_variance-Residual_variance_cluster-Residual_variance_subject;

Proportion_cluster=Residual_variance_cluster/Total_variance;

Proportion_explained=Variance_explained/Total_variance;

Proportion_subject=Residual_variance_subject/Total_variance;

Residual_ICC=Residual_variance_cluster/(residual_variance_cluster+residual_variance_subject);

run;

title 'Total variance';

proc print data=outcome label noobs;

label variation_coefficient="coefficient of variation" total_variance="total variance" proportion_explained="explained" proportion_subject="subject" proportion_cluster="cluster";

format proportion_cluster proportion_explained proportion_subject percent10.1;

var variation_coefficient total_variance proportion_cluster proportion_explained proportion_subject;

run;

title 'Residual variance';

proc print data=outcome label noobs;

label residual_icc="residual ICC";

format residual_icc percent10.1;

var Residual_ICC;

run;

data _null_;

call symput ("var_tot_cluster", ".");

call symput ("var_tot_res", ".");

call symput ("var_res_cluster", ".");

call symput ("var_res_res", ".");

call symput ("coeff_var", ".");

run;

%END;

%IF &type=dich %THEN %DO;

proc glimmix data=&data method=laplace; /*first we compute the model including both within and between effects*/

class &cluster ;

model &score =&predictor &between &macropred/s dist=binomial;

random &cluster;

ods output ParameterEstimates=fixed;

run;

data fixed;

set fixed;

effect=upcase(effect);

run;

%let n=%sysfunc(countw(&predictor)); /* now we separate the between from the wihtin effect*/

%do var=**1** %to &n;

%let withinvar= %qscan (&predictor, &var);

data _null_;

set fixed;

if effect="&withinvar" then call symput ('estimate', estimate);

run;

%put &estimate;

data &data;

set &data;

coeff=&estimate;

part_var_&var = &withinvar * coeff;

run;

%end;

data &data;

set &data;

within=sum(of part_var_1-part_var_&n);

drop coeff part_var_1-part_var_&n;

run;

proc glimmix data=&data method=laplace;

class &cluster ;

model &score= &macropred/offset=within dist=binomial s; /* we continue with only the within effect, as an offset*/

random intercept /sub=&cluster solution; /* the random intercept captures also the between effect of the predictor*/

output out=lp pred(noblup)=lp;/* we save the linear predictor as we will need this to computed the explained variance*/

ods output CovParms=cluster_resid solutionr=RI; /* write variances to total_cluster_resid*/

run;

proc means data=lp var; /*we compute the variance of the linear predictor. This is the variance explained by the model. Note that we do not need to fit a full and empty model here, as was the case with the continuous model*/

where lp ne **0**; /* sas puts the lp to 0 if the cluster has a missing value. Since these are not used for the computation of the variances in the multilevel model, we don't use them for the variance of the fixed part either*/

var lp;

output out=var_lp var=var_lp;

run;

data _null_;

set var_lp;

if _type_=**0** then call symput ("var_explained", var_lp);

run;

data _null_;

set cluster_resid;

if CovParm="Intercept" then call symput ("var_cluster", Estimate);

run;

proc means data=&data; /*we compute the prevalence to quantify the total variance*/

var &score;

output out=prev mean=prev;

run;

data _null_;

set prev;

if _type_=**0** then call symput ("prevalence", prev);

run;

data outcome; /* we compute the variance partitioning measures of interest*/

prevalence=&prevalence;

Residual_variance_cluster=&var_cluster;

pi=constant("PI");

Residual_variance_subject=pi*pi/**3**;

Variance_explained=&var_explained;

Total_variance=Residual_variance_cluster+Residual_variance_subject+variance_explained;

Proportion_cluster=Residual_variance_cluster/Total_variance;

Proportion_explained=Variance_explained/Total_variance;

Proportion_subject=Residual_variance_subject/Total_variance;

Residual_ICC=Residual_variance_cluster/(residual_variance_cluster+residual_variance_subject);

run;

title 'Total variance';

proc print data=outcome label noobs;

label total_variance="total variance" proportion_explained="explained" proportion_subject="subject" proportion_cluster="cluster";

format prevalence proportion_cluster proportion_explained proportion_subject percent10.1;

var prevalence total_variance proportion_cluster proportion_explained proportion_subject;

run;

title 'Residual variance';

proc print data=outcome label noobs;

label residual_icc="residual ICC";

format residual_icc percent10.1;

var Residual_ICC;

run;

data _null_;

call symput ("var_cluster", ".");

call symput ("var_explained", ".");

run;

%END;

%IF &type=ord %THEN %DO;

proc glimmix data=&data method=laplace; /*first we compute the model including both within and between effects*/

class &cluster ;

model &score =&predictor &between &macropred/ link=cumlogit dist=multinomial s;

random &cluster;

ods output ParameterEstimates=fixed;

run;

data fixed;

set fixed;

effect=upcase(effect);

run;

%let n=%sysfunc(countw(&predictor)); /*we compute the within-effect*/

%do var=**1** %to &n;

%let withinvar= %qscan (&predictor, &var);

data _null_;

set fixed;

if effect="&withinvar" then call symput ('estimate', estimate);

run;

%put &estimate;

data &data;

set &data;

coeff=&estimate;

part_var_&var = &withinvar * coeff;

run;

%end;

data &data;

set &data;

within=sum(of part_var_1-part_var_&n);

drop coeff part_var_1-part_var_&n;

run;

proc glimmix data=&data method=laplace; /* we continue with only the within effect, as an offset*/

class &cluster ;

model &score= &macropred/offset=within link=cumlogit dist=multinomial s;

random intercept /sub=&cluster solution;

output out=lp pred(noblup)=lp;

ods output CovParms=cluster_resid solutionr=RI; /* write variances to total_cluster_resid*/

run;

data lp_sort; set lp; run;

proc sort data=lp_sort; by _level_;run;

proc means data=lp_sort var;

where lp ne **0** and _level_=**1**; /* sas puts the lp to 0 if the cluster has a missing value. Since these are not used for the computation of the variances in the multilevel model, we don't use them for the variance of the fixed part either. We use level one. This is arbitrary but the variance is identical for all levels.*/

var lp;

output out=var_lp var=var_lp;

run;

data _null_;

set var_lp;

if _type_=**0** then call symput ("var_explained", var_lp);

run;

data _null_;

set cluster_resid;

if CovParm="Intercept" then call symput ("var_cluster", Estimate);

run;

data outcome;

Residual_variance_cluster=&var_cluster;

pi=constant("PI");

Residual_variance_subject=pi*pi/**3**;

Variance_explained=&var_explained;

Total_variance=Residual_variance_cluster+Residual_variance_subject+variance_explained;

Proportion_cluster=Residual_variance_cluster/Total_variance;

Proportion_explained=Variance_explained/Total_variance;

Proportion_subject=Residual_variance_subject/Total_variance;

Residual_ICC=Residual_variance_cluster/(residual_variance_cluster+residual_variance_subject);

run;

title 'Total variance';

proc print data=outcome label noobs;

label total_variance="total variance" proportion_explained="explained" proportion_subject="subject" proportion_cluster="cluster";

format proportion_cluster proportion_explained proportion_subject percent10.1;

var total_variance proportion_cluster proportion_explained proportion_subject;

run;

title 'Residual variance';

proc print data=outcome label noobs;

label residual_icc="residual ICC";

format residual_icc percent10.1;

var Residual_ICC;

run;

data _null_;

call symput ("var_cluster", ".");

call symput ("var_explained", ".");

run;

%END;

**%MEND** variance_partition;
